# Prediction of Rheological Properties of PVA Fiber and Nano-SiO_2_-Reinforced Geopolymer Mortar Based on Back Propagation Neural Network Model Optimized by Genetic Algorithm

**DOI:** 10.3390/polym17081046

**Published:** 2025-04-12

**Authors:** Guo Zhang, Peng Zhang, Juan Wang, Shaowei Hu

**Affiliations:** 1 School of Water Conservancy and Transportation, Zhengzhou University, Zhengzhou 450001, China; zhangguo@stu.zzu.edu.cn (G.Z.); wangjuan@zzu.edu.cn (J.W.); hushaowei@zzu.edu.cn (S.H.); 2 State Key Laboratory of Tunnel Boring Machine and Intelligent Operations, Zhengzhou 450001, China

**Keywords:** geopolymer mortar, rheology, back propagation, genetic algorithm, prediction

## Abstract

The rheological properties of mortar are of vital importance to ensure the quality and durability of engineering structures, improving construction efficiency and adapting to different construction environments. This research focused on examining the rheological properties of geopolymer mortar (GM) with the incorporation of metakaolin (MK), nano-SiO_2_ (NS) and polyvinyl alcohol (PVA) fibers. The research focused on varying concentrations of PVA fiber ranging from 0 to 1.2% (interval of 0.2%) and NS ranging from 0 to 2.5% (interval of 0.5%). As the mix proportion optimization of GM is normally carried out experimentally, a significant amount of labor and material resources was consumed. Based on large amounts of authentic operation data, a prediction model of rheological properties for NS- and PVA-fiber-reinforced GM was developed using a back propagation (BP) neural network. Subsequently, the parameters were refined using a genetic algorithm (GA) to predict the rheological properties of GM reinforced with different dosages of NS and PVA fiber. Three rheological parameters, including static yield stress, plastic viscosity and dynamic yield stress, were used to evaluate the rheological properties of GM. Moreover, parameters of Root Mean Square Error (RMSE), Mean Absolute Percentage Error (MAPE) and Mean Absolute Error (MAE) were applied to assess the capability of the algorithms. When the GA–BP neural network was used, compared with the BP neural network, the coefficient of determination (*R*^2^) of the static yield stress, plastic viscosity, and dynamic yield stress increased by 4.40%, 2.11% and 15.28%, respectively, and the GA–BP neural network provided a superior fitting effect, higher prediction accuracy and faster convergence. Based on the outputs of the developed model, the GA–BP neural network can be adopted as a precise method to forecast the rheological properties of GM reinforced with NS and PVA fibers.

## 1. Introduction

In the global economy, the building industry is one of the biggest consumers of raw materials, energy, and natural resources [1]. Cement-based materials are prevalent in the construction industry worldwide [2]. For each ton of cement, approximately 1 kg of sulfur dioxide (SO_2_), 810 kg of carbon dioxide (CO_2_), 10 kg of dust and 2 kg of nitrogen oxides (NO_x_) are generated [3,4]. Consequently, traditional cement production is a high-energy, costly in terms of resources and environmentally burdensome industry. Finding cost-effective and ecologically friendly alternatives to cement production is crucial to solving the growing environmental problems [5,6]. The most promising bonding material is geopolymer [7], which can be used in place of or in addition to traditional cement. Geological materials and industrial wastes, including MK, blast furnace slag, bottom ash and fly ash (FA), can be used to create geopolymer [8,9]. The production of geopolymer results in minimal emissions of CO_2_, NO_x_ and SO_x_ [10]. A significant advancement in the use of geopolymer in garbage fixation was realized by Khalil [11]. Additionally, the mechanical properties of GM are either equal to or better in comparison to those found in traditional cement mortar [12]. Compared to bulk-FA mortar and traditional silicate cements, Sate et al. [13] discovered that lignite ash GM had higher compressive strength and resistance to sulphate attack. As a result, GM, which has enormous potential to address problems related to pollution, is a fantastic alternative to conventional cement mortar.

GM has a short setting time and high early strength, which is suitable for the preparation of repair and reinforcement materials. Although GM is superior in many aspects, steps must still be taken to enhance the properties of a construction with a harsh service environment and high-performance requirements. To enhance the densification of GM and thus improve the rheological properties [14,15], durability [16,17] and mechanical properties [18,19,20], researchers have incorporated NS into geopolymers in recent years. In the opinion of Alomayri et al. [21], NS could improve both the microstructure and the mechanical properties of GM by creating refined interfacial zones and dense matrices. Zidi et al. [22] found that adding a reasonable amount of NS increased the mechanical strength of GM. As in-depth research on nanoparticles has progressed and the cost of manufacturing nanoparticles has continuously decreased [23], nanoparticles have developed a broad prospect for application in GM as an admixture.

The tensile and flexural strength of GM is relatively low. Studies have shown that adding specific fibers, such as PVA fibers, steel fibers [24] and polypropylene fibers [25], can increase the durability and toughness of GM. The great tensile strength, elastic modulus and affordability [26,27] have made PVA fibers one of the more advanced green building materials of the future [28]. Xu et al. [29] analyzed the mechanical properties and mix fraction of PVA-fiber-reinforced GM based on fly ash with two distinct PVA fiber lengths. Tanyildizi and Yonar [30] explored the way high temperatures affected the mechanical properties of GM reinforced with PVA fibers in the same area of study. The results of the study demonstrated that raising the fiber proportion can enhance the flexural and compressive strengths of the mortar. To enhance the properties of GM, some researchers are currently attempting to combine PVA fiber with NS. Through experimental studies, Zhang et al. [31] assessed the ideal blend of PVA fiber and NS in GM and argued about the combined effects. The application of NS and PVA fiber to enhance the durability and mechanical properties of eco-friendly geopolymers was studied by Xu et al. [32]. Some researchers have also shown that the mixing of PVA fibers and NS boosts the bonding properties of GM [33]. Therefore, optimizing the proportions and properties of PVA-fiber- and NS-reinforced GM is crucial for subsequent experiments and future construction.

The relationship between the mixture design and the rheological properties of GM is intricate [34], non-linear and dependent on several variables. Furthermore, the preparation process raises the cost of the finished product by consuming more material, time and labor. Based on these reasons, it is important to utilize machine learning to predict the rheological properties of GM [35,36]. In order to examine the intricate and nonlinear interactions between the mix proportion and the material properties, neural networks have recently been extensively used in material property research and prediction. Artificial neural networks were used by Bagheri et al. [37] to develop a function of the compressive strength of boroaluminosilicate geopolymers. The *R*^2^ was 0.95, and the RMSE was 0.07, showing the excellent accuracy and dependability of the model. Awoyera et al. [38] employed neural networks and genetic programming to predict the strength properties of self-compacting geopolymer. In response, parameters for flexural strength, split-tensile strength and compressive strength were used to create the smallest error-prediction model. Nevertheless, there is a lack of research on the rheological properties of NS- and PVA-fiber-reinforced GM and no appropriate prediction methods or models for predicting the relationship between the rheological properties and mix proportion.

In addition to utilizing neural network models, there are also several multifactor traditional models based on various polynomials. The traditional models do not need to be pre-trained. The necessary prediction accuracy can be achieved by choosing the order of the approximate polynomial within a given range of parameter values. Neural network models have significant advantages in dealing with complex nonlinear relationships and high-dimensional data, but the training process is complex, and the interpretation is poor. Traditional multifactor models perform well in achieving simplicity and interpretability but are sensitive to the data and have a limited generalization ability. In practical application, the appropriate model should be selected according to the requirements of specific tasks and data characteristics. Considering the complexity of mix proportions, a neural network model is selected to predict them in this study.

Therefore, a method to predict the rheological properties of NS- and PVA-fiber-reinforced GM will be proposed by using the BP neural network in this research. Utilizing the BP neural network, complex nonlinear models can be constructed, and a good nonlinear mapping ability is demonstrated [39]. The initial weights and thresholds of the input, hidden and output layers of the BP neural network are very uncertain during operations given that they are tied to the initial data selection. Additionally, the BP neural network is characterized by a slow convergence rate [40], and it is prone to becoming trapped in the local minima during the operation. To solve those problems, the mix proportion of NS- and PVA-fiber-reinforced GM can be optimized using a genetic algorithm (GA). The thresholds, weights, learning rules and structure of the GA–BP algorithm are logical and have been demonstrated to outperform a conventional BP neural network in terms of prediction. In conclusion, a GA–BP neural network can be applied not only for forecasting the rheological properties but also for assisting engineers and researchers in the mixture design of NS- and PVA-fiber-reinforced GM.

## 2. Experiment Program

Predicting the rheological properties of GM via mix proportion is the aim of this study. The controlled variable approach was employed to carry out the experimental design of the mix proportion. Only the PVA fiber and NS content as main factors should be varied, while the cement/sand ratio, water/cement ratio and water glass modulus should have fixed values.

Table 1 and Table 2 list the properties of PVA and NS fibers. Table 3 displays the XRF-determined chemical composition and component amounts of MK and FA. The high-efficiency water-reducing agent was a polycarboxylate superplasticizer, which had a water-reducing rate of 21%. Through trial mixing, the mixture with 30% FA, 70% MK, a cement/sand ratio of 1.0 and a water-to-binder ratio of 0.65 was established. It has been shown that the mechanical and physical properties of mortar are improved when FA is substituted with MK [41]. Applying the water glass modulus calculations and adjustments suggested by other researchers [42], sodium hydroxide was added to lower the modulus of water glass from 3.2 to 1.3. By introducing an appropriate quantity of water, the sodium oxide mass fraction reaches 15%.

This research conducted rheological property tests on GM to evaluate the rheological properties when incorporated with NS and PVA fibers. Static yield stress is the initial resistance of a material from rest to flow, which affects stability and maneuverability at rest. The dynamic yield stress reflects the minimum stress required to maintain the flow and is related to the continuity and thixotropy of the flow, which affects the application effect and efficiency. Plastic viscosity indicates resistance in the flow process and affects construction performance and material stability. These indicators comprehensively consider the performance of materials at rest, flow and different application conditions and are an important basis for evaluating rheological properties. In consequence, three indicators, including plastic viscosity, static yield stress and dynamic yield stress, were evaluated as rheological parameters [43]. The lower static yield stress and plastic viscosity are helpful for improving the fluidity and construction efficiency of mortar. The low dynamic yield stress helps the mortar to remain uniform during the flow process, reducing delamination and segregation. At the same time, the high static yield stress and the appropriate plastic viscosity help to maintain the structural stability of the mortar in the static state, prevent the mortar from cracking during the curing process and improve the durability. Appropriate dynamic yield stress can enhance adaptability under different shear rates and reduce structural damage during construction. Therefore, rheological parameters have an important effect on the workability and durability of GM.

Operating a neural network requires the utilization of a defined quantity of training data. The data utilized for this study came from references [44,45] and were analyzed as indicated in Table 4. Referring to the commonly used PVA fiber contents in traditional concrete, PVA fiber contents of 0.2, 0.4, 0.6, 0.8, 1.0 and 1.2% were used in this study [46]. A low PVA fiber ratio (such as 0–1.2%) ensures that key mechanical parameters such as Young’s modulus and elongation at break of the material reach an ideal balance. Therefore, it is necessary to further study a broader range of PVA fiber content to obtain the optimal threshold. Similarly, through consulting relevant information [47] and trial preparation, the contents of NS are determined to be 0.5%, 1.0%, 1.5%, 2.0% and 2.5%.

Compared with the abovementioned references, this study combined the effects of changes in mono-doped NS or PVA fiber content and the effects of changes in mixed NS and PVA fiber content on rheological properties. On this basis, the GA–BP neural network model is trained by the experimental data. Finally, the rheological parameters are predicted by the mix proportion. When the value of the additive is not in the training area, the prediction accuracy may be reduced due to the lack of corresponding training data, which is a normal phenomenon. According to the current research, it is not meaningful to study the mix proportion of the mortar outside the training area. There is no denying that this study can further promote the study of the rheological properties of NS- and PVA-fiber-reinforced GM.

## 3. Model Establishment

In general, the task of this study is investigating the nonlinear relationship between the amount of admixture (such as NS and PVA fiber) and the performance index (rheological parameters), aiming to minimize the prediction error. The architecture of a BP neural network consists of an input layer, several hidden layers and an output layer. GAs are employed to enhance the predictive accuracy of the BP neural network. This is achieved by encoding operational parameters as genetic elements and optimizing these parameters through genetic operations like selection, crossover and mutation. Ultimately, the process establishes a correlation between the mix proportions and rheological parameters, enabling the prediction of rheological properties.

### 3.1. BP Neural Network

**Figure 1:**
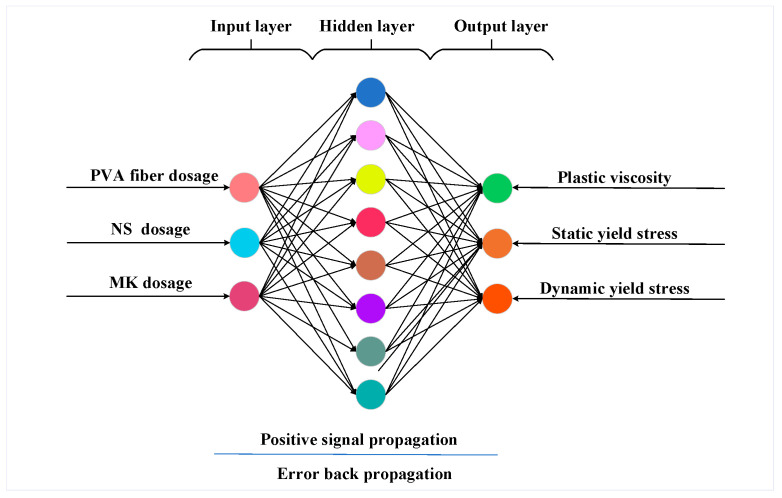
The structure of the BP neural network.

The quantity of neurons depends on a particular problem analysis in each layer of the BP neural network. Interconnected nodes or neurons make up the BP neural network. These nodes accept input, process data through mathematical computations and produce output signals [48,49]. Neurons are interconnected by weighted connections that confirm the intensity of the signal transmitted among neurons. NS will substitute FA with an equivalent mass and concurrently alter the quantity of MK added. Therefore, three material parameters, including NS, PVA fiber and MK content, that impact the rheological properties of GM are ultimately selected as input parameters. If the input functions of the neural network demonstrate a multicollinear problem, it will lead to limited modeling ability and overfitting on the training set. Considering that the ratio between the content of FA and MK is certain with a correlation coefficient of 1, only the content of MK is taken as one of the input parameters. Three neurons, including plastic viscosity, static yield stress and dynamic yield stress make up each output-layer neural network. Theoretically, any complex nonlinear mapping can be handled by a three-layer BP neural network. The BP neural network is capable of fulfilling most predictive requirements with a hidden layer. Eight neurons are used in this investigation. During the training phase, seventy percent of the sample data was used for training while the thirty percent served as the prediction set. The topology is indicated in Figure 1.

A BP neural network is characterized by forward information propagation and a backward error feedback mechanism, based on a gradient descent to find adaptation. Equations (1) and (2) [50] provide the adjustment ways for weights $w_{ij}$ and biases $b_{j}$.

(1) $$w_{ij}=w_{ij}-\eta_{1}\frac{\partial E\left(w,b\right)}{\partial w_{ij}}=w_{ij}-\eta_{1}\delta_{ij}x_{i}$$

(2) $$b_{j}=b_{j}-\eta_{2}\frac{\partial E\left(w,b\right)}{\partial_{bj}}=b_{j}-\eta_{2}\delta_{j}$$

where E is the error function, $\eta_{1}$ is the learning rate of the weights, $\eta_{2}$ is the learning rate of the biases, $x_{i}$ represents the node output values, $w_{ij}$ represents the weights and $b_{j}$ represents the biases.

The output of every neuron is calculated using an activation function based on the weighted sum of its inputs. The rectified linear unit (ReLU) function, the hyperbolic tangent function and the sigmoid function are the most often utilized activation functions [51].

The sigmoid function is defined by Equation (3):

(3) $$y\left(x\right)=\frac{1}{1+e^{-x}}$$

The hyperbolic tangent function is defined by Equation (4):

(4) $$y\left(x\right)=\tanh(x)=\frac{2}{{1+e}^{-2x}}-1$$

The ReLU function is defined by Equation (5):

(5) $$y(x)=\left\{\begin{array}{c} \max(0,x),x\geq 0\\ 0,x<0 \end{array}\right.$$

where y is the function, x is the input parameter.

The ReLU function is used in the modeling of this study. When the input is positive, the ReLU function is different from the sigmoid and tanh functions, which have a gradient saturation problem. Additionally, the ReLU function only contains linear relationships and is computed more quickly than sigmoid and tanh. The output layer uses the purelin transfer function [52] and the hidden layer selects the tansig activation function. The trainlm algorithm is a gradient descent method employed for backpropagation training.

On account of the topology structure of the neural network, let the output value of the i−th node in the input layer be expressed as $x_{i}$ and the total number of nodes be n. For the j−th node in the hidden layer, the input value is denoted as $h_{j}$, the output value as $h_{j}^{\prime }$ and the number of nodes is m.

(6) $$h_{j}=\sum_{i=1}^{n}(w_{ji}x_{i})+b_{j}$$

where $w_{ji}$ is the connection weight between the i−th node of the output layer, the j−th node of the hidden layer and $b_{j}$ is the threshold of the j−th node for the hidden layer.

(7) $$h_{j}^{\prime }=\varphi_{1}(h_{j})=\frac{2}{1+e^{-2h_{j}}}-1$$

where $\varphi_{1}(x)$ is the activation function of the hidden layer, specifically the hyperbolic tangent tansig transfer function. The input value of the k−th node in the output layer is represented by $u_{k}$ and the output value is represented by $u_{k}^{\prime }$.

(8) $$u_{k}=\sum_{j=1}^{m}(w_{kj}h_{j}^{\prime })+b_{k}$$

where $w_{kj}$ represents the connection weight between the j−th node in the hidden layer, the k−th node for the output layer and $B_{k}$ signifies the threshold of the output layer node.

(9) $$u_{k}^{\prime }=\varphi_{2}(\mu_{k})=u_{k}$$

where $\varphi_{2}(x)$ is the activation function of the output layer, which is the purelin linear transfer function.

In this study, the random number generation method is used to initialize the weight matrix. The method of least squares is employed to refine the weights of the network and rebuild the weight function. To prevent overfitting, an L_2_ regularization term is incorporated, thereby enhancing the capability of the model to generalize. Finally, the weight is updated again. Considering that the BP neural network undergoes training and learning, the discrepancy between the predictive outputs and the practical results diminishes. In the fit method of the BP neural network, the scipy.optimize.least_squares function is used for optimization, which internally uses the Levenberg–Marquardt algorithm or other numerical optimization algorithms instead of the traditional gradient descent algorithm. Therefore, the quantity of iterations and the learning rate are not set directly. The setting functions of the BP neural network are listed in Table 5.

### 3.2. The Genetic Algorithm

**Figure 2:**
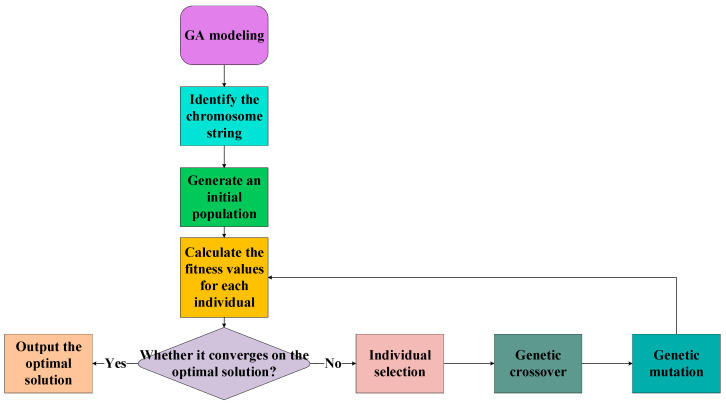
The process flowchart of a GA.

Inspired by natural selection and evolution, GAs are an approach for population-based optimization that can solve challenging search problems [53]. Chromosomes made up of several genes encode possible solutions to a problem in a GA. A population of chromosomes is created at random at the beginning of the GA. The ratio of fitness to cost function is used to determine the level of fitness of each chromosome. Chromosomes are more probable to be selected with higher fitness for reproduction compared to those with lower fitness. Parts of two or more preexisting chromosomes are joined during crossover to produce children. In order to keep chromosomes from becoming trapped in a local optimum, mutation can occur in certain chromosomes, which involves making tiny and random changes to chromosomes [54]. Until the stop condition is satisfied, GA carries out multiple iterations of crossover, mutation and reproduction [55]. In the end, the GA will output a set of optimal weight configurations for the individual with the highest fitness score. The major parameters of a GA are described in Table 6 and the process flowchart of a GA is indicated in Figure 2.

Here are the procedural steps involved in GA optimization.

(1) Define the fitness function. The ability to acquire data using just the objective function of a problem is one feature of a GA. The assessment of individual fitness in minimization problems reflects the objective function [56], which is indicated in Equation (10).

(10) $$F=C_{\alpha }-f$$

where $C_{\alpha }$ is a constant, f means objective function and F represents the fitness function.

(2) Individual selection. Superior individuals are chosen, and inferior individuals are eliminated through the selection process. The next generation inherits those who are physically fit. The proportionate fitness scaling approach, one of the traditional selection decision methods, determines the selection probability of an individual depending on their fitness value [57], displayed in Equation (11).

(11) $$P_{i}=\frac{f_{i}}{\sum_{j=1}^{n}f_{j}}$$

where n is the population size and f is the fitness value of an individual within the population.

(3) Genetic crossover. Crossover is the course of creating a new individual through the replacement and recombination of genetic material from two parent individuals. The specific encoding design needs to be taken into account while creating the crossover operator. A common crossover operator in continuous optimization approaches is simulated binary crossover (SBX), and the chosen gene modifications adhered to Equations (12) and (13).

(12) $$y_{i}^{1}=0.5[(1-\beta )x_{i}^{1}+(1+\beta )x_{i}^{2}]$$

(13) $$\beta \left(u\right)=\left\{\begin{array}{c} (2u)\frac{1}{n_{c}+1},\frac{4u}{n_{c}}u\leq 0.5\\ 2(1-u))\frac{1}{n_{c}+1},\mathrm{or} \end{array}\right.$$

where u is an evenly distributed random number within the interval [0, 1], y represents the alteration of the selected gene and x is the parent individual.

(4) Genetic mutation. Introducing mutations preserves individual variation within the population while simultaneously enhancing local search capabilities [58]. The most straightforward mutation method is uniform mutation, which substitutes an evenly distributed random number within a specific range for the initial gene value of each gene, shown in Equation (14).

(14) $$x_{k}^{\prime }=U\min+r(U\max-U\min)$$

where r is an evenly distributed random number in the region of [0, 1].

### 3.3. GA–BP Neural Network

The principle of a GA to optimize the parameters of a BP neural network is to simulate the natural selection process and use the global search ability to avoid the BP network falling into the local optimal. The GA first randomly generates an initial population, with each individual representing a set of network parameters. Excellent individuals are selected through fitness assessments (such as prediction error), and new individuals are generated through crossover and mutation operations to increase population diversity. After many generations of evolution, the optimal parameter combination, which is found by the GA, is passed to the BP neural network. Therefore, the BP neural network can start training with better initial parameters, thus improving the convergence speed, reducing the prediction error and enhancing the generalization ability and prediction accuracy of the model.

**Figure 3:**
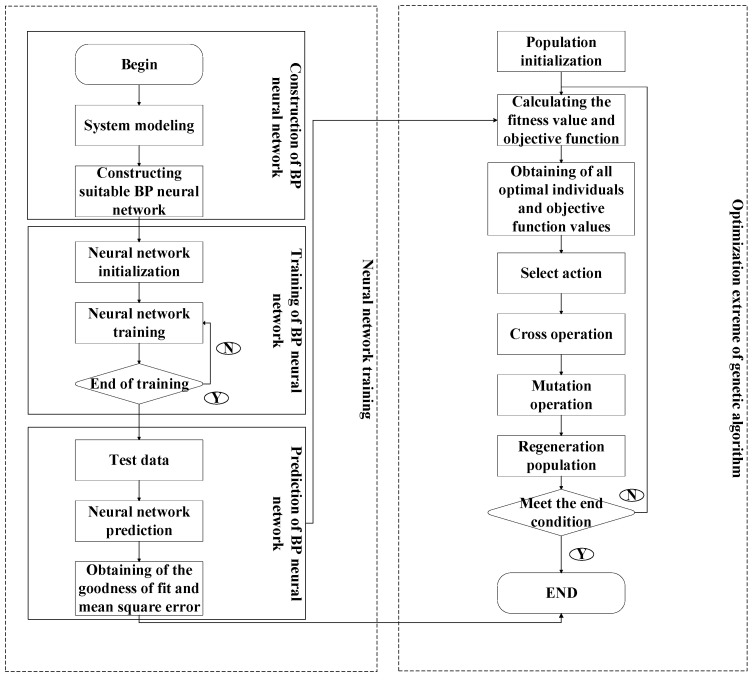
Prediction model based on the GA–BP neural network.

The GA–BP neural network offers a novel approach to optimization issues by combining the learning and approximation capabilities of a BP neural network with the global search and optimization capabilities of a GA. A GA initially encodes the weights and thresholds of a neural network. The weights and biases of the neural network are initialized, and the parameters of the GA are set, including population size, crossover probability and mutation probability. The genetic method enhances the weights and biases of neural networks. The network weights and thresholds are progressively refined by selection, crossover and mutation. The latest codes are produced continuously. The goal is to identify an approximate optimal solution by exploring a large number of potential solution spaces. The GA–BP neural network is trained by the BP neural network based on the network structure and initial values identified by the GA. The weight and bias are modified to get the output of the neural network closer to the true value. The processes of GA optimization and BP neural network training are iterated until the stopping criteria are satisfied or the highest quantity of iterations is achieved. The flow diagram for the entire prediction model is displayed in Figure 3.

The GA–BP neural network model exhibits a number of noteworthy benefits in practical applications. Because of the powerful global search capabilities of GAs, neural networks can more effectively converge to the global optimum and avoid getting trapped in local optima. The GA–BP neural network, combining the benefits of GAs and BP neural networks, has great versatility and supports a variety of network architectures in terms of scale and kind. The GA–BP neural network handles complex data more effectively and has some resilience to noisy data and outliers. Moreover, the GA–BP neural network can eliminate manual intervention, reach intelligent optimization and automatically modify the structure and factors of neural networks. Through parallel computation, the GA–BP neural network is capable of accelerating both the training and prediction phases, which is particularly appropriate for complicated model structures and huge data sets.

## 4. Discussion

The model is trained using seventy percent of the sample data, which is determined by the research methodology and training model. Following model training, testing was conducted using the remaining thirty percent of the sample dataset, and the test results were examined and assessed.

### 4.1. Normalization and Correlation Analysis

The specific purpose of normalization is to create a statistical distribution of the uniform sample. Employing initial statistics for evaluation can complicate the testing process, as individual data points might cause difficulties, perhaps leading to a network convergence failure or an extension of the training duration. The sample data must be normalized [59] prior to additional data processing and analysis. Normalization helps eliminate systematic errors due to differences in factor magnitude and calculation errors due to inconsistent data units [60].

(15) $$x_{\left(m,i\right)}^{\prime }=\frac{x_{\left(m,i\right)}-x_{m,\min}}{x_{m,\max}-x_{m,\min}}$$

(16) $$y_{\left(n,i\right)}^{\prime }=\frac{y_{\left(n,i\right)}-y_{n,\min}}{y_{n,\max}-y_{n,\min}}$$

where $x_{\left(m,i\right)}$ represents the content of component m in the mix proportion i, which ranges from 1 to 16. m ranges from 4 to 6, which corresponds to MK, PVA fiber and NS, respectively. $x_{m,\min}$ is the minimum parameter across different mix proportions and $x_{m,m\mathrm{ax}}$ is the maximum parameter. $x_{\left(m,i\right)}^{\prime }$ is the normalized rheological parameters of mix proportion i. $y_{\left(n,i\right)}$ is the content of component n in mix proportion i, which ranges from 1 to 16. n ranges from 8 to 10, which corresponds to plastic viscosity, dynamic yield stress and static yield stress. $y_{n,\min}$ is the minimum parameter with different mix proportions and $y_{n,\max}$ is the maximum parameter. $y_{\left(n,i\right)}^{\prime }$ is the normalized rheological parameters for mix proportion i.

**Figure 4:**
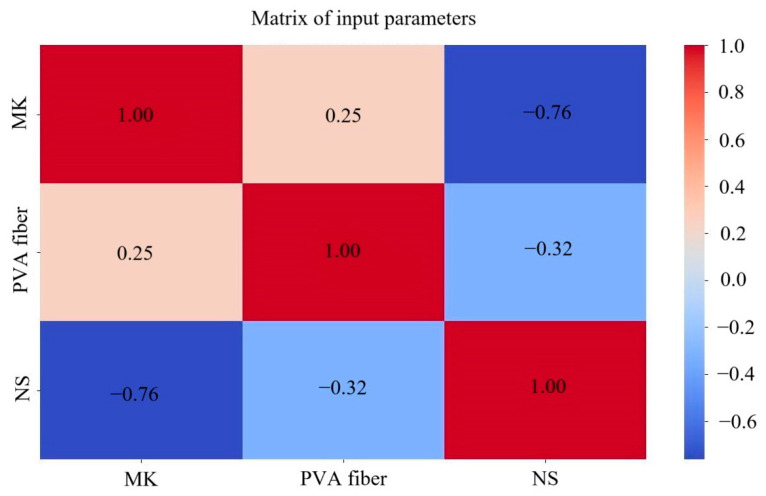
Matrix diagram of correlation coefficients.

When two or more correlated variables are analyzed, correlation analysis is used to determine the extent to which the variables are related based on the analysis findings. The correlation coefficient is either a high positive or high negative value when the input parameters have a significant correlation. This phenomenon leads to low model efficiency or difficulty explaining the process by which input parameters affect output parameters. Consequently, the correlation between NS, PVA fiber and MK content should be analyzed first before training with the neural network. The analysis findings are shown in Figure 4, which indicates that the identical input parameters have a correlation coefficient of 1. The correlation coefficient between NS and MK was 0.76, the strongest correlation, while the correlation coefficient between PVA fiber and MK was 0.25, the lowest. To summarize, the correlation coefficients of the three input parameters were all less than 0.8, suggesting that they were unrelated to one another. Considering the rheological properties of PVA-fiber- and NS-reinforced GM were predicted using MK, NS and PVA fiber content as input values, there is no multicollinearity problem.

A total of 16 experimental datasets were utilized to train both the BP neural network and the GA–BP neural network. The remaining six sets of data were then predicted using the trained neural networks, while the predictions and actual results were compared and evaluated.

### 4.2. Experimental Results and Analysis

To verify the learning and prediction capabilities of the BP neural network model enhanced by a GA, the experimenter ran the code associated with the neural network model and automatically completed the network training, parameter optimization, error calculation and graphical output. In the training set, the content of NS varied from 0% to 2.5%. In the test set, in order to analyze the accuracy of the model prediction, the content of NS was fixed and only the content of PVA fiber was changed. It can be concluded from previous studies that the larger the PVA fiber content is, the larger the rheological parameters are. The PVA fiber content ranges from 0% to 1.2%. Therefore, NS was fixed at 1% of the optimal content in the study, and PVA content was changed to help test the fitting effect of the model. Table 7 indicates the test set data.

**Figure 5:**
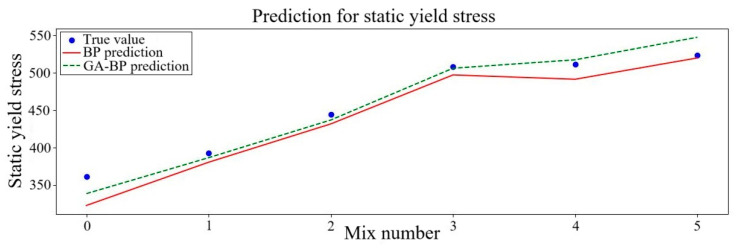
Static yield stress predicted results of neural networks.

**Figure 6:**
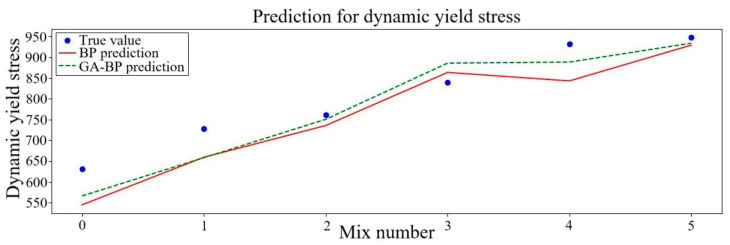
Dynamic yield stress predicted results of neural networks.

**Figure 7:**
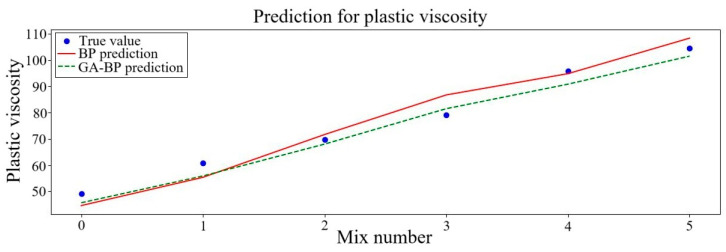
Plastic viscosity predicted results of neural networks.

Three rheological parameters, including plastic viscosity, dynamic yield stress and static yield stress predicted from the two different kinds of neural networks, were contrasted with the outcomes of the experiment. The comparison findings are displayed in Figure 5, Figure 6 and Figure 7. To more clearly observe the variation tendency of the data and the degree of prediction fitting, six sets of data were selected. In these sets of data, the fixed NS content was set at 1% while the PVA fiber content was multiplied from 0.2% to 1.2%. The outcomes of the experiment are consistent with the projected trend.

The yield stress of the mortar gradually increases as the PVA fiber content increases, as seen in Figure 5 and Figure 6. Compared with existing experimental studies, upon reaching a PVA fiber content of 1.2%, the dynamic yield stress and static yield stress predicted decrease by 1.9% and 0.7% in the BP neural network, while they change by 1.4% and 4.5% in the GA–BP neural network. The rheological parameters of geopolymer mortar were added with the increase in PVA fiber volume content ranging from 0% to 1.2%. These findings mostly concur with those of earlier research. The surface of PVA fiber is covered in many hydroxyl hydrophilic functional groups [61] and is prone to water accumulation, which lowers the amount of free water that serves as a lubricant between particles. As a consequence, the initial flow speed of the mortar causes the particle spacing to decrease, increasing flow resistance. Further analysis indicates that when the PVA fiber content is small, the numerical trends predicted by the two neural networks are gradually increasing and relatively similar. When the fiber content of PVA is 0.8–1.2%, both the BP and GA–BP neural networks predicted that the PVA fiber content first decreased and then recovered, and the prediction trend of the GA–BP neural network is smoother. The above illustrates that GA–BP neural network prediction will be more stable and will not be excessively disturbed by data. It also suggests the impact on yield stress tends to be flat when PVA fiber content increases to a certain range.

Figure 7 displays the impact of PVA fiber content on the plastic viscosity of GM. The viscosity of plastic steadily adds as the PVA fiber content keeps increasing. Compared to the real data, upon reaching a PVA fiber content of 1.2%, the plastic viscosity predicted increases by 3.6% in the BP neural network, while it decreases by 2.8% in the GA–BP neural network. At the same time, the predicted rheological parameters of geopolymer mortar are increasing in the same way as the experimental results. This further indicates the accuracy and reliability of the forecast results. PVA fiber clumping and tangling become apparent. The likelihood of overlap and the quantity of mesh structures among the fibers introduce additional flow resistance, leading to an increase in plastic viscosity [62]. The main elements that contribute to the production of net structures are the hydration flocculation and interparticle forces within cement mortar. From the prediction curve, the deviation of prediction values of the BP neural network and the GA–BP neural network is the largest when the PVA content is 0.8%. The predicted values of the BP and GA–BP neural networks are both greater than the experimental results, which may be due to some errors in the measurement of experimental data.

It is normal that there are some deviations between the prediction results of the two neural network models and the experimental results. The neural network model relies on a large amount of data for training, and the number of training sets in the study is relatively small, so the accuracy of the model needs to be improved. At the same time, during the corresponding test, there may be some errors in the test data due to improper operation and other reasons. In the subsequent research, the amount of test data should be further increased to improve the robustness of the model and reduce the test error. Considering that the optimization algorithm has certain randomness, multiple predictions can be made, and the average value of the predicted results is taken. Through the above methods, the deviation between the predicted results and the test results can be further reduced.

The predictive capability of the enhanced artificial neural network is confirmed using the test dataset. Several statistical error analysis metrics are employed as benchmarks to evaluate the model, thereby offering a more comprehensive and robust comparison [63]. The *RMSE* measures the size of the model prediction error, the *MAPE* measures the relative size of the model prediction error, the *MAE* measures the average size of the model prediction error and the *R*^2^ measures the degree of fit of the model. Therefore, *RMSE*, *MAPE*, *MAE* and *R*^2^ as the evaluation indicators were chosen in accordance with evaluation principles and practices.

*RMSE*:

(17) $$RMSE=\sqrt{\frac{\sum_{i=1}^{n}\left|o_{i}-t_{i}\right|}{n}}$$

*MAPE*:

(18) $$MAPE=\frac{\sum_{i=1}^{n}\left|\frac{o_{i}-t_{i}}{o_{i}}\right|\times 100\%}{n}$$

*MAE*:

(19) $$MAE=\frac{\sum_{i=1}^{n}\left|o_{i}-t_{i}\right|}{n}$$

*R*^2^:

(20) $$R^{2}=1-\frac{\sum_{i=1}^{n}{\left(o_{i}-t_{i}\right)}^{2}}{\sum_{i=1}^{n}\left(o_{i}-\bar{o_{i}}\right)}$$

In the above three equations, $\bar{o_{i}}$ is the average of the true values, $o_{i}$ is the true value, $t_{i}$ is the model-predicted value and n is the sample size.

The size of the prediction error is estimated by the *RMSE* [64], and the lower the value, the more accurate the predictions of the model are. Since the *RMSE* is a square root and has the same units as the original data, it can be readily interpreted. The *MAPE* represents the error as a percentage and is sensitive to outliers because the error is calculated in proportion to the actual value. The *MAE* provides the average size of the prediction error. *MAE* is less sensitive to outliers than *RMSE* because it does not involve squaring operations. The capacity of the model to explain data variability and its prediction accuracy both increase with the proximity of the *R*^2^ value to 1. It reflects how closely the predictive values of the neural network align with the practical values but does not directly reflect the size of the error [65]. An analysis of the evaluation metrics for the neural networks presents the outcomes in Table 8.

Among the three rheological parameters, the largest fitting error is the dynamic yield stress. According to the graph, except for the PVA content of 0.8%, the predictive value is smaller than the practical value and the difference is large. The greatest stress necessary to maintain the mortar flowing is known as the dynamic yield stress [66]. The numerical value of dynamic yield stress is high, and the test error is relatively large. Therefore, it is normal for the error to be larger than other rheological parameters. From the perspective of rheological properties, the three rheological coefficients increase with the increase in PVA content. The average *R*^2^ is about 0.86 in the BP neural network and 0.91 in the GA–BP neural network, which meet the prediction accuracy requirements. Both the *RMSE* and *MAE* values are error values presented on account of the size of the initial data, and the error range of the fitted data relative to the real data needs to be further analyzed. The *MAPE* value of both neural networks is no more than 7.0%, which is within the allowable range. Properly improving the accuracy of the fitting model can make the prediction result more accurate. Once the mix ratio is determined, the corresponding rheological parameters can be obtained, and a clear mix ratio optimization direction can be obtained. However, the limitations of artificial specimen production in the test process should be taken into account. In the process of research, it is not necessary to pursue the accuracy of the fitting model too much. Considering the limited measurements of the actual measuring tools, excessive model accuracy is not necessary. Based on the accuracy analysis of the two models, the GA–BP neural network outperforms the BP neural network.

The GA–BP neural network model can better fit the data by optimizing the initial weight and bias through a genetic algorithm, thus increasing the *R*^2^ and reducing the error value. The higher the *R*^2^, the faster the convergence rate. In summary, the GA–BP neural network effectively overcomes the limitations of the standard BP model by combining the global optimization ability of genetic algorithms and the local optimization ability of BP neural networks. Additionally, the GA–BP neural network has better global search ability, convergence speed, generalization ability and robustness. The results mentioned above are consistent with those of earlier research on the GA–BP network [67,68]. A comparison of the expected values showed that the GA–BP neural network-based predicted values for the rheological parameters of NS- and PVA-fiber-reinforced GM are consistent with the real circumstances.

Compared with traditional experimental methods, the GA–BP neural network can significantly reduce labor and material costs when studying rheological properties of geopolymer mortar. The traditional method requires a large number of experiments to determine the rheological parameters corresponding to the mix proportion, which is time-consuming and costly. The GA–BP model can predict rheological properties quickly and accurately based on existing data and reduce the number of experiments and material waste. At present, the input layer of the model is only set to three, and the input range can be expanded according to the specific research direction in the subsequent study. At the same time, the model can be further optimized in the future so that the optimal mix proportion can be automatically screened to reduce manual intervention and improve efficiency. Considering that the laboratory conditions cannot fully fit the actual conditions of the project, a lot of tests should be carried out on the construction site based on the theoretical mix proportion to get the best project mix proportion. The GA–BP model can make predictions based on existing test data, reduce unnecessary test times and improve efficiency. In consequence, the GA–BP neural network can possibly provide guidance on the study of the rheological properties and engineering applications of NS- and PVA-fiber-reinforced GM.

## 5. Conclusions

In this study, BP and GA–BP networks are employed to anticipate the effects of NS and PVA fiber on the rheological parameters of GM. Here are the major conclusions.

(1) Through correlation analysis, the correlation coefficients of NS, PVA fiber and MK content are all less than 0.8, so those three parameters are unrelated to one another. Consequently, there will not be a multicollinearity problem when predicting the rheological properties of NS- and PVA-fiber-reinforced GM using these three factors as input parameters.
(2) The findings of the analysis show that there is a concordance between the experimental and predicted rheological parameters for the rheological parameters of NS- and PVA-fiber-reinforced GM. The *R*^2^ of the plastic viscosity, dynamic yield stress and static yield stress increased by 2.11%, 15.28% and 4.40%, respectively, when the GA–BP neural network was employed instead of the BP neural network. As a result, the predictive capacity of the GA–BP neural network outperforms the BP neural network.
(3) The GA–BP neural network can simplify the prediction of rheological properties for NS- and PVA-fiber-reinforced GM by capturing the complex nonlinear mappings between the rheological properties and the mix proportions of the mortar. Furthermore, this model can effectively optimize the mix proportions of NS- and PVA-fiber-reinforced GM, whose predictions could be employed in composite mix proportion testing to enhance the efficiency of the testing process.

## Figures and Tables

**Table 1:** Properties of NS.

Loss on Ignition (%)	pH	Stacking Density (g/cm^3^)	Nominal Particle Size (nm)	Specific Surface Area (m^2^/g)
1.0	6.21	0.054	30	200

**Table 2:** Properties of PVA fibers.

Elongation at Fracture (%)	Filament Diameter (µm)	Fiber Length (mm)	Flexural
6.5	40	12	1560

**Table 3:** Chemical composition of MK and FA.

Composition	Ingredient Contents(wt.%)							
	K_2_O	Al_2_O_3_	Na_2_O	TiO_2_	MgO	SiO_2_	Fe_2_O_3_	CaO
MK	0.26	43.52	0.18	0.06	0.35	54.34	1.17	0.42
FA	2.91	23.05	0.97	1.45	1.16	55.47	5.75	5.17

**Table 4:** Mixture proportions of GM.

Mix Number	NaOH	Water Glass	Fly Ash (FA)	Metakaolin (MK)	PVA Fiber	Nano Particle	Water Reducer	Static Yield Stress	Dynamic Yield Stress	Plastic Viscosity
	kg/m^3^	kg/m^3^	kg/m^3^	kg/m^3^	%	%	kg/m^3^	Pa	Pa	Pa·s
M-0-0	71	445.4	184.1	429.5	0	0	3.07	350.72	577.6	42.8
P-0.2-0	71	445.4	184.1	429.5	0.2	0	3.07	365.169	653.5	57.5
P-0.4-0	71	445.4	184.1	429.5	0.4	0	3.07	404.755	759.5	65.4
P-0.6-0	71	445.4	184.1	429.5	0.6	0	3.07	497.123	843.1	79.6
P-0.8-0	71	445.4	184.1	429.5	0.8	0	3.07	513.444	912.3	84.1
P-1.0-0	71	445.4	184.1	429.5	1.0	0	3.07	522.628	924.8	99.7
P-1.2-0	71	445.4	183.1	427.2	1.2	0	3.07	547.441	950.9	111.9
N-0-0.5	71	445.4	182.2	425	0	0.5	3.07	336.83	563.6	42.1
N-0-1.0	71	445.4	181.2	422.7	0	1.0	3.07	296.202	472.8	40.9
N-0-1.5	71	445.4	180.2	420.4	0	1.5	3.07	358.185	569.2	43.8
N-0-2.0	71	445.4	182.2	425	0	2.0	3.07	372.596	634.1	47.1
N-0-2.5	71	445.4	182.2	425	0	2.5	3.07	394.126	708.2	52.6
PN-0.2-1.0	71	445.4	182.2	425	0.2	1.0	3.07	361.484	631.4	49.1
PN-0.4-1.0	71	445.4	182.2	425	0.4	1.0	3.07	392.677	728.7	60.9
PN-0.6-1.0	71	445.4	182.2	425	0.6	1.0	3.07	444.476	760.7	69.7
PN-0.8-1.0	71	445.4	183.1	427.2	0.8	1.0	3.07	507.826	839.3	79.1
PN-1.0-1.0	71	445.4	181.2	422.7	1.0	1.0	3.07	511.162	931.9	95.9
PN-1.2-1.0	71	445.4	180.2	420.4	1.2	1.0	3.07	523.544	947.4	104.5
PN-0.6-0.5	71	445.4	182.2	425	0.6	0.5	3.07	469.699	802.9	75.9
PN-0.6-1.5	71	445.4	180.2	420.4	0.6	1.5	3.07	535.874	938.2	82.8
PN-0.6-2.0	71	445.4	182.2	425	0.6	2.0	3.07	585.459	976.9	91.7
PN-0.6-2.5	71	445.4	180.2	420.4	0.6	2.5	3.07	683.382	995.5	109.6

**Table 5:** The setting functions of the BP neural network and the quantity of neurons in each layer.

Parameters	Output Layer Neurons	Output Layer Transfer Function	Hidden Layer Transfer Function	Hidden Layer Neurons	Input Layer Neurons
Value	3	Purelin	Tansig	8	3

**Table 6:** The main parameters of a GA.

Parameters	Number of Iterations	Mutation Probability	Individual Number	Crossover Probability
Value	100	0.001	600	1

**Table 7:** The test set data.

Mix Number	Metakaolin (MK)	PVA Fiber	Nano Particle	Static Yield Stress	Dynamic Yield Stress	Plastic Viscosity
	kg/m^3^	%	%	Pa	Pa	Pa·s
1	425	0.2	1.0	361.484	631.4	49.1
2	425	0.4	1.0	392.677	728.7	60.9
3	425	0.6	1.0	444.476	760.7	69.7
4	427.2	0.8	1.0	507.826	839.3	79.1
5	422.7	1.0	1.0	511.162	931.9	95.9
6	420.4	1.2	1.0	523.544	947.4	104.5

**Table 8:** Comparative analysis of evaluation metrics for neural network models.

Rheological Parameters	Neural Network Models	*R* ^2^	*RMSE*	*MAPE*	*MAE*
Static yield stress	BP	0.91	19.53	3.86%	16.18
	GA–BP	0.95	14.16	2.58%	11.25
Dynamic yield stress	BP	0.72	60.17	6.80%	52.02
	GA–BP	0.83	47.48	5.49%	41.53
Plastic viscosity	BP	0.94	4.61	5.88%	4.07
	GA–BP	0.96	3.64	4.77%	3.41

## Data Availability

The original contributions presented in this study are included in the article. Further inquiries can be directed to the corresponding author.
